# Isolation of Extended-Spectrum *β*-lactamase- (ESBL-) Producing *Escherichia coli* and *Klebsiella pneumoniae* from Patients with Community-Onset Urinary Tract Infections in Jimma University Specialized Hospital, Southwest Ethiopia

**DOI:** 10.1155/2018/4846159

**Published:** 2018-12-13

**Authors:** Mengistu Abayneh, Getnet Tesfaw, Alemseged Abdissa

**Affiliations:** ^**1**^ School of Medical Laboratory Sciences, Mizan-Tepi University, Mizan Aman, Ethiopia; ^2^School of Medical Laboratory Sciences, Institute of Health Sciences, Jimma University, P.O. Box 378, Jimma, Ethiopia

## Abstract

**Background:**

*Klebsiella pneumoniae* and *Escherichia coli* are the major extended-spectrum *β*-lactamase- (ESBL-) producing organisms increasingly isolated as causes of complicated urinary tract infections and remain an important cause of failure of therapy with cephalosporins and have serious infection control consequence.

**Objective:**

To assess the prevalence and antibiotics resistance patterns of ESBL-producing *Escherichia coli* and *Klebsiella pneumoniae* from community-onset urinary tract infections in Jimma University Specialized hospital, Southwest Ethiopia, 2016.

**Methodology:**

A hospital-based cross-sectional study was conducted, and a total of 342 urine samples were cultured on MacConkey agar for the detection of etiologic agents. Double-disk synergy (DDS) methods were used for detection of ESBL-producing strains. A disc of amoxicillin + clavulanic acid (20/10 *µ*g) was placed in the center of the Mueller–Hinton agar plate, and cefotaxime (30 *µ*g) and ceftazidime (30 *µ*g) were placed at a distance of 20 mm (center to center) from the amoxicillin + clavulanic acid disc. Enhanced inhibition zone of any of the cephalosporin discs on the side facing amoxicillin + clavulanic acid was considered as ESBL producer.

**Results:**

In the current study, ESBL-producing phenotypes were detected in 23% (*n* = 17) of urinary isolates, of which *Escherichia coli* accounts for 76.5% (*n* = 13) and *K. pneumoniae* for 23.5% (*n* = 4). ESBL-producing phenotypes showed high resistance to cefotaxime (100%), ceftriaxone (100%), and ceftazidime (70.6%), while both ESBL-producing and non-ESBL-producing isolates showed low resistance to amikacin (9.5%), and no resistance was seen with imipenem. In the risk factors analysis, previous antibiotic use more than two cycles in the previous year (odds ratio (OR), 6.238; 95% confidence interval (CI), 1.257–30.957; *p* = 0.025) and recurrent UTI more than two cycles in the last 6 months or more than three cycles in the last year (OR, 7.356; 95% CI, 1.429–37.867; *p* = 0.017) were found to be significantly associated with the ESBL-producing groups.

**Conclusion:**

Extended-spectrum *β*-lactamases- (ESBL-)producing strain was detected in urinary tract isolates. The occurrence of multidrug resistance to the third-generation cephalosporins, aminoglycosides, fluoroquinolones, trimethoprim-sulfamethoxazole, and tetracyclines is more common among ESBL producers. Thus, detecting and reporting of ESBL-producing organisms have paramount importance in the clinical decision-making.

## 1. Introduction

Drug-resistant microbes of all kinds can move among people and animals, from one country to another without notice. Since 21st century, it is thought that the emergence of extended-spectrum *β*-lactamase- (ESBL-) producing bacteria may present an increasing risk of transmission of resistant strains in humans and animals. They is a worrying global public health issue as infections caused by such enzyme-producing organisms are associated with a higher morbidity and mortality and greater fiscal burden. The problem is clearly severe in developing countries where studies on this subject, drug availability, and its appropriate use were limited and resistance rate was high [1, 2].

ESBL-producing organisms are capable of hydrolyzing penicillin, broad-spectrum cephalosporins, and monobactams, but they do not affect the cephamycins or carbapenems, and their activity is inhibited by clavulanic acid. In addition, ESBL-producing organisms are frequently exhibiting resistance to other antimicrobial classes such as fluoroquinolones, aminoglycosides, and trimethoprim-sulfamethoxazole due to associated resistance mechanisms, which may be either chromosomally or plasmid-encoded [3–6]. The widespread use of third-generation cephalosporin was believed to be the major cause of mutations in these enzymes that leads to the emergence of plasmid-encoded ESBLs. These ESBLs were transferred between bacteria by plasmids, which were in turn spread by clonal distribution between hospitals and countries through patient mobility [7].

The presence of ESBLs complicates antibiotic selection, especially in patients with serious infections, such as bacteremia. The reason for this is that ESBL-producing bacteria, including those originating in the community, are often multiresistant to various antibiotics; an interesting feature of isolates that produce CTX-M (CTX stands for cefotaximases and M for Munich) is the coresistance to the fluoroquinolones. Type CTX-M ESBLs have been described as an enzyme preferentially hydrolyzing cefotaxime over ceftazidime and also hydrolyzing cefepime with high efficiency [8, 9].

The spread and the burden of ESBL-producing bacteria are greater in developing countries. Findings of a recent review showed that pooled prevalence of healthcare-associated infections in resource-limited settings (15.5%) was twice the average prevalence in Europe (7.1%). Some plausible reasons for this difference include the following conditions that are prevalent in low-income countries: crowded hospitals, more extensive self-treatment and use of nonprescription antimicrobials, poorer hygiene in general and particularly in hospitals, and less effective infection control [10–12].

In comparison with the rest of the world, there is generally a lack of comprehensive data regarding ESBL-producing Enterobacteriaceae in African countries. The real situation of antibiotic resistance is also not clear since ESBL-producing organisms as well as non-ESBL producers are not routinely cultured and their resistance to antibiotics cannot be tested. Therefore, this study was conducted to determine the prevalence and antimicrobial resistance pattern of ESBL-producing *Escherichia coli* and *Klebsiella pneumoniae* isolated from community-onset UTI patients at Jimma University Specialized Hospital, Southwest Ethiopia.

## 2. Materials and Methods

### 2.1. Study Area and Period

The study was conducted at Jimma University Specialized Hospital (JUSH) in Jimma town from March to June, 2016. Jimma University specialized hospital is located southwest of Addis Ababa, capital city of Ethiopia, and currently it is the only more than 300-bedded teaching hospitals in the Southwestern part of the country.

### 2.2. Study Design and Study Participants

A cross-sectional study was conducted to evaluate the prevalence and antimicrobial resistance pattern of ESBL-producing *E. coli* and *K. pneumoniae* among community-onset UTI infections in Jimma University Specialized hospital (JUSH), Southwest Ethiopia. All outpatients with age groups of ≥15 years and who are suspected of symptoms of urinary tract infections as diagnosed clinically within 48 hours of admission and those coming from outpatient departments for laboratory diagnosis of urine were taken as study participants. Written informed consent was obtained from the patients or guardians of the patient before data collection. The patients who were suspected of symptoms of UTI were identified by communicating with the physicians as he/she put “UTI” on the laboratory request forms as identification numbers for those patients suspected of UTI as diagnosed clinically and requested to laboratory for urinalysis tests. Patients who received antibiotics within the past 2 weeks were excluded.

### 2.3. Definitions

*Community-onset infections* are defined infections that have an onset within 48 hours of hospital admission or that present in the outpatient setting [13]. Such infections can be divided into two groups. The first group is associated with healthcare institutions and includes patients receiving intravenous treatment or specialized care, those received hemodialysis treatment or antineoplastic chemotherapy, those who have attended at any hospital clinic within the previous 30 days, those who have been admitted in an acute care center two days within the previous 90 days, and residents of nursing homes or long-term care centers. The second group represents truly community-acquired infections in patients who do not meet the above mentioned criteria.

### 2.4. Data Collection

#### 2.4.1. Sociodemographic and Clinical Data

Sociodemographic and other clinical data such as age, sex, previous antibiotic use more than two cycles per year, previous intravenous therapy at home or any clinics and repeated outpatient visits at hospital in the last 30 days, previous hospitalization in an acute care center 2 or more days in the 90 days, previous invasive procedures of the urinary tract, previous wound care by specialized nursing or family within 30 days, presence of diabetes mellitus, and recurrent urinary tract infections were collected by face-to-face interviewing of the patient or guardian of the patient by using a well-structured questionnaire before laboratory sample collection.

#### 2.4.2. Laboratory Data Collection

A total of 342 midstream urine samples were collected with a sterile, wide mouthed, and leak proof containers. A 10 *µ*l (0.01 ml) well-mixed urine sample was inoculated into MacConkey agar (Oxoid, UK) and incubated at 37°C for 24 hours. The colony count with at least 10^5^ CFU/ml for single midstream urine was taken as positive urine culture as described previously [14]. All the isolates were preliminarily screened by their colony morphology, pigment production (pink to colorless flat or mucoid colonies), and Gram-staining techniques (Gram-negative rods, nonsporing, and noncapsulated). Further identifications of isolates were made by conformation of motility and other relevant biochemical tests. For example, an isolate was considered as *E. coli* when it is indole (dark pink ring) and methyl-red positive, citrate negative (no change or remained green) and urea negative, gas and acid producer, and motile and were considered as *K. pneumoniae* when it is indole and methyl-red negative, citrate positive, urea slow producing, and nonmotile. In case of delay, the isolated bacteria were kept at 2–8°C in the nutrient broth for not more than 24 hrs until the antimicrobial sensitivity test was done.

### 2.5. ESBL Detection Methods

ESBL-producing *E. coli and K. pneumonia* were first screened for ESBL production by the phenotypic method and then will be confirmed by the phenotypic confirmatory test as per Clinical and Laboratory Standards Institute (CLSI) guidelines 2014 [15].

#### 2.5.1. Phenotypic Screening for ESBL Production

The ESBL screening test was performed by the standard disk diffusion method by using ceftazidime (30 *µ*g), cefotaxime (30 *µ*g), and ceftriaxone (30 *µ*g) (Oxoid, UK). More than one antibiotic disc were used for screening to improve the sensitivity of ESBLs detection, as recommended by CLSI guidelines 2014 [15]. Freshly grown colonies were suspended into normal saline, and the turbidity of the suspension was adjusted at 0.5 McFarland's standard. This suspension was inoculated onto Mueller–Hinton agar (Oxoid, UK) with sterile cotton swab, and then all the above three antibiotics discs were placed at a gap of 20 mm and incubated at 35 ± 2°C for 16–18 hours. The isolates with reduced susceptibility to cefotaxime (zone diameter of ≤27 mm), ceftazidime (zone diameter of ≤22 mm), and ceftriaxone (zone diameter of ≤25 mm) around the disks were suspected as ESBLs producers [15].

#### 2.5.2. Phenotypic Confirmation of ESBL Producers

Confirmation of suspected ESBLs producers was done by using the double-disk approximation or double-disk synergy (DDS) method on Mueller–Hinton agar, as recommended by CLSI guidelines 2014 [15]. A disc of amoxicillin + clavulanic acid (20/10 *µ*g) was placed in the center of the Mueller–Hinton Agar plate, and then cefotaxime (30 *µ*g) and ceftazidime (30 *µ*g) were placed at a distance of 20 mm (center to center) from the amoxicillin+ clavulanic acid disc on the same plate. The plate was incubated at 37^o^C for 24 hours and examined for an enhancement or expansion of inhibition zone of the oxyimino-*β*-lactam caused by the synergy of the clavulanate in the amoxicillin-clavulanate disk which was interpreted as positive for ESBL production.

### 2.6. Antimicrobial Susceptibility Testing

The antimicrobial susceptibility testing was done by using Kirby–Bauer disc-diffusion technique on Mueller–Hinton agar according to the CLSI guidelines 2014 [15] for the following antimicrobial discs: amoxicillin/clavulanic acid (20/10 *µ*g), cefotaxime (30 *µ*g), ceftriaxone (30 *µ*g), ceftazidime (30 *µ* g), ampicillin (10 *µ*g), cephalothin (30 *µ*g), ciprofloxacin (5 *µ*g), nalidixic acid (30 *µ*g), norfloxacin (10 *µ*g), gentamycin (10 *µ*g), amikacin (30 *µ*g), tetracycline (30 *µ*g), trimethoprim-sulfamethoxazole (1.25/23.75 *µ*g), imipenem (30 *µ*g), and chloramphenicol (30 *µ*g) (Oxoid; UK). The selections of antimicrobial agents depend on the availability and recommendations from CLSI 2014 [15]. After overnight incubation of the Mueller–Hinton agar plate with antimicrobial discs at 37°C, the zone of inhibition was measured by using a ruler and interpreted by comparing the Kirby–Bauer chart. Control strains *(K. pneumoniae* ATCC 700603 and *Escherichia coli* ATCC 25922) were used to monitor quality of antibiotic discs during antimicrobial susceptibility testing and during ESBL detection methods.

*Multidrug resistance (MDR)* is defined as resistance to three or more classes of antibiotics [16].

### 2.7. Data Analysis

The data were analyzed by using SPSS version 16.0. The difference in categorical variables and susceptibility pattern between ESBL producer and non-ESBL-producing groups were analyzed statistically by using chi-squared (Fisher's exact) test. Odds ratios (ORs) and their 95% confidence intervals (CIs) were calculated, and *p* value <0.05 was regarded as statistically significant. The findings were presented in tables.

## 3. Results

### 3.1. Clinical Specimens and Isolates Recovered

In the current study, about 74 (21.6%) of urine samples were confirmed as positive urine culture, of which 63 (85.1%) were *E. coli* and 11 (14.9%) were *K. pneumoniae*. Out of 74 positive urine cultures, 17 (23.0%) were confirmed as positive for ESBL production. *E. coli* accounts for large number of urinary isolates as well as higher proportion of ESBL production (13 (76.5%)) than *K. pneumoniae* (4 (23.5%)). The maximum bacterial isolates and higher proportion of ESBL-producing strains were isolated from females (12 (70.6%)) than male (5 (29.4%)). The mean age of patients from which ESBL producers was detected is 35.07 years (±13.30 SD). From the total ESBL producers, 9 (52.9%) were isolated from patients older than 50 years of age (Table 1).

Patients in the ESBL group were further divided into healthcare-associated and community-acquired groups, and 9 (52.9%) of ESBL isolates were isolated from individuals who have no history of healthcare contact (community acquisition), with a higher proportion of *E. coli*, 8 (61.5%) (Table 1).

### 3.2. Risk Factors for Isolations of ESBL-Producing Strains

In the current study, different types of possible risk factors were analyzed but only any antibiotic use more than two cycles in the previous year (OR = 6.238; 95% CI = 1.257–30.957; *p* = 0.025) and recurrent UTI more than two cycles in the last 6 months or more than three cycles in the last year (OR = 7.356; 95% CI = 1.429–37.867; *p* = 0.017) were identified as an independent risk factors for acquisition of ESBL-producing strains (Table 2).

### 3.3. Resistance Profile of ESBL-Producing and Non*-*ESBL-Producing Isolates

In the current study, ESBL-producing isolates showed higher resistance not only towards third-generation cephalosporins but also towards other antimicrobial agents tested (*p* = 0.001). Resistance rates to cefotaxime, ceftriaxone, and ceftazidime are 100%, 100%, and 70.6%, respectively. All ESBL-producing and non-ESBL-producing isolates were resistant to ampicillin (Table 3).

### 3.4. Resistance Profiles of Isolates from Healthcare-Associated versus Community-Acquired

The current study finding showed that there are no differences in resistance profiles of isolates from patients who have history of healthcare-associated infections and those isolates from pure community-acquired infections for most of antimicrobial agents tested (*p* > 0.05) (Table 4).

### 3.5. Multidrug Resistance Pattern of *E. coli* and *K. pneumoniae*

In this study, multidrug resistance (≥3 antibiotic classes) pattern was more prevalent among the ESBL-producing isolates. In our finding, 82.4% of ESBL-producing isolates were showed cross-resistance against both co-trimoxazole and tetracycline, with 52.9% coresistant to tetracycline, fluoroquinolones, co-trimoxazole, aminoglycosides, and chloramphenicol classes of antibiotics plus beta-lactam groups of antibiotics. Coexistence of ESBL phenotype with 5, 6, and 7 types of non-*β* lactam antibiotics were 11 (64.7%), 9 (51.9%), and 4 (23.5%), respectively (Table 5).

## 4. Discussion

Until recently, ESBL-producing organisms were viewed as hospital-acquired or healthcare-associated pathogens, i.e., affecting patients who had typically been in hospitals or other healthcare facilities. However, in recent years, ESBL-producing Enterobacteriaceae isolates have shifted from the hospital to the community or have been recognized in community patients who had no prior contact with the healthcare system [17].

In our study, the ESBL-producing phenotype were detected in 23% (17/74) of the urinary isolates, which was slightly higher than a result obtained in Taiwan (20.7%) [18] and higher than previous study finding in the same area, in which the proportions of ESBL producers from outpatients were 14.3% [19]. The higher finding in our study may be related with the more distributions of ESBL from time to time in the study area.

In contrast, the proportions of ESBLs observed in our study are lower than that in previous reports in Saudi Arabia (42.38%) [20] and in Tanzania (45.2%) [21]. The reasons for this decline observed in our study may be explained by the inclusion of only single specimens from outpatient only. This reason supported by the fact that hospital environment played a role for maintenance of ESBL-producing organism [22]. Moreover, higher rate of fecal carriage of ESBLs producers among inpatients were also observed elsewhere in Saudi Arabia [23], which support the notions that hospital acquired isolates are more likely to become ESBL producer.

Although advanced molecular methods for species identifications and characterizations of ESBL typing were not conducted in our study, *Escherichia coli* accounts for a large number of urinary isolates as well as higher numbers of ESBL production 76.5% than *K. pneumoniae* 23.5%. Our finding was correlated with the previous study finding in the same area, in which three (75%) of the four ESBL producers from outpatients were *E. coli* [19]. Another study finding in Israel also showed that higher prevalence of ESBL-producing isolates from outpatients was for *E. coli* (57.8%) [8].

A community-origin explaining this rise of ESBLs has been observed in many surveys, but in our setting it is difficult to ascertain accurately, as faecal colonization surveys among humans without direct or indirect hospital exposure are scarce. Accordingly, the gut plays a prominent role in the development of antibiotic resistance and the emergence of resistant microorganisms which may be subsequent agents of urinary infection in vulnerable patients [24, 25]. A recent report from Cameroon [26] showed 16% fecal carriage of ESBLs isolates with the majority (over 80%) of these being *E. coli*, and in Saudi Arabia 12.7% isolates were ESBLs producers, of which 95.6% were *E. coli* and 4.4% were *K. pneumoniae* [27]. Therefore, in patients admitted to the hospital with community-acquired UTIs, the risk factors for acquiring ESBL-producing organisms should be considered before initiating treatment.

In our study, 52.9% of ESBL-producing isolates were isolated from individuals who have no history of healthcare contact (community-acquisition), with a higher proportion of *E. coli*, 61.5%. This finding is in agreement with the previous report done in Switzerland [28], where 64% patients with ESBL-producing *E. coli* had community-acquired and 36% had healthcare-associated UTIs, and in Spain 68% had community-acquired and 32% cases comprised healthcare-associated cases [29].

The data concerning risk factors for the development of infection with ESBL-producing bacteria among outpatients are very scarce in our settings. In the our study, any antibiotic use more than two cycles in the previous year (OR = 6.238; 95% CI =1.257–30.957; *p*=0.025) and recurrent UTI more than two cycles in the last 6 months, or more than three cycles in the last year (OR = 7.356; 95% CI =1.429–37.867; *p*=0.017) were identified as independent risk factors for development or acquisition of ESBL-producing organisms. Our finding is also correlated with the previous studies in Israel [8]. This may suggest that the greater exposure to antibiotics may lead to development of selection pressures.

In our study finding, 82.4% of ESBL-producing isolates showed multidrug resistance to different families of antibiotics such as SXT and tetracycline. This finding is correlated with other studies in developing countries such as Tanzania [21], and in Guinea-Bissau [30], nearly all ESBL-producing *E. coli* isolates in the community were multidrug-resistant.

Multidrug resistance nature of these isolates may be explained by the fact that ESBLs are plasmid-mediated enzymes which are carrying multiresistant genes by plasmid, transposon, and integron and also they are readily transferred to other bacteria, not necessarily of the same species, and bacteria with multiple resistances to antibiotics are widely distributed in hospitals and increasingly being isolated from community [31]. This fact supported by recent surveys from Canada [32] and Spain [33] has illustrated an alarming trend of associated resistance among ESBL-producing organisms isolated from community sites, especially those producing CTX-M types, which exhibited coresistance to SXT, tetracycline, gentamicin, and ciprofloxacin. Thus, our study results well support the fact that ESBL producers confer high levels of resistance to not only third-generation cephalosporins but also to other non-*β* lactams group of antibiotics.

This study also revealed that all ESBL-producing and non-ESBL-producing isolates showed resistance to ampicillin. Our finding correlated with the fact that *β*-lactamase-negative isolates may be resistant to ampicillin by other mechanisms. In contrast, better susceptibility was noticed to amikacin and no resistance was observed with imipenem. Better susceptibility to amikacin was also noticed in previous study in our study area [19]. This may be explained by the absence of routine use of amikacin as empirical therapy and its absence of considerable cross-resistance with *β*-lactam groups of antibiotics.

Although MIC determination of resistant strains was not conducted in our study, further analysis of the antimicrobial resistance pattern among isolates from infections associated with healthcare institutions and those from “true” community-acquired infections showed that there are no differences between the two groups in the resistance pattern (*p* ≥ 0.05). The similarity in resistance pattern between healthcare associated isolates and “true” community-acquired isolates may be related to frequent use and misuse of non-prescribed antibiotics in the community as well as in healthcare facilities especially in private healthcare sectors and the time to time spreads of resistant strains from healthcare institutions to the community in our setting. Therefore, this finding gives an attention from health policymakers to promote rational use of antibiotics in healthcare settings as well as in the community.

## 5. Conclusion

The data obtained in our study indicate that ESBL-positive phenotypes were prevalent not only patients who had typically been in hospitals or other healthcare facilities but also in community patients. Coresistance to other classes of antimicrobial agents such as aminoglycosides, fluoroquinolones, co-trimoxazole, and tetracyclines was also more common among ESBLs positive phenotypes. Any antibiotic use more than two cycles in the previous year and recurrent UTI was identified as independent risk factors for acquisition of ESBL-producing organisms. Thus, our finding gives an attention to promote rational use of antibiotics in healthcare settings and surveillance studies in order to monitor the changes in the antimicrobial resistance pattern.

### 5.1. Limitations of the Study

Advanced molecular methods for species identification and characterization of ESBL typing and MIC determination of resistant strains were not conducted due to lack of availability.

## Figures and Tables

**Table 1:** Distribution of ESBL-producing and non-ESBL-producing *E. coli* and *K. pneumoniae* isolated from community-onset urinary tract infections in JUSH, Southwest Ethiopia, 2016.

Characteristics		Total isolate *N* (%)	ESBLs-positive (*n* = 17)	ESBLs-negative (*n* = 57)	*p* value
Age and sex groups					
** **Age	15–49	51 (68.9%)	8 (47.1%)	43 (75.4%)	0.130
	≥50	23 (31.1%)	9 (52.9%)	14 (24.6%)	
** **Sex	Female	53 (71.6%)	12 (70.6%)	41 (71.9%)	0.874
	Male	21 (28.4%)	5 (29.4%)	16 (28.1%)	

Organisms					
** ** *E. coli*		63 (85.1%)	13 (76.5%)	50 (87.7%)	0.263
** ** *K. pneumonia*e		11 (14.9%)	4 (23.5%)	7 (12.3%)	

Distribution					
** **Community-acquired		49 (66.2%)	9 (52.9%)	40 (70.2%)	0.192
** **Healthcare-associated		25 (33.8%)	8 (47.1%)	17 (29.8%)	

**Table 2:** Characteristics of patients infected with ESBLs-producing and non-ESBLs-producing *E. coli* and *K. pneumoniae* among community-onset urinary tract infections in JUSH, Southwest Ethiopia, 2016.

Variables	Category	ESBLs*-*positive	ESBLs*-*negative	OR (95% CI)	*p* value
Age groups	15–49	8 (47.1%)	43 (75.4%)		0.130
	≥50	9 (52.9%)	14 (24.6%)		
Sex	Female	12 (70.6%)	41 (71.9%)		0.874
	Male	5 (29.4%)	16 (28.1%)		
*Healthcare-associated risk factors*					
Any antibiotic use more than two cycles in the previous year	Yes	13 (76.5%)	27 (47.37%)	6.238 (1.257–30.957)	0.025^*∗*^
	No	4 (23.5%)	30 (52.63%)			
Prior intravenous therapy at home or any clinic within 30 days	Yes	3 (17.6%)	7 (12.3%)		0.608
	No	14 (82.4%)	50 (87.7%)		
Repeated outpatient visit or attendant at hospital within 30 days	Yes	2 (11.76%)	10 (17.5%)		0.829
	No	15 (88.23%)	47 (82.5%)		
Previous hospitalization in an acute care center >2 days within 90 days	Yes	1 (5.9%)	1 (1.8%)		0.532
	No	16 (94.1%)	56 (98.2%)		
History of invasive procedure of the urinary tract within the previous year	Yes	0	1 (1.8%)		0.966
	No	17 (100%)	56 (98.2%)		
Previous wound or specialized nursing care within 30 days	Yes	1(5.9%)	1 (1.8%)		0.495
	No	16 (94.1%)	56 (98.2%)		
*Underlying diseases*					
Presence of diabetes mellitus	Yes	5 (29.4%)	9 (15.8%)		0.815
	No	12 (70.6%)	48 (84.2%)		
Recurrent UTI >two cycle in the last 6 months or > three cycles in the last year	Yes	7 (41.2%)	8 (14.0%)	7.356 (1.429–37.867)	0.015^*∗*^
	No	10 (58.8%)	49 (86.0%)		

OR: odds ratio, CI: confidence interval, ^*∗*^*p* value less than 0.05.

**Table 3:** Resistance profiles of ESBL-producing and non-ESBL-producing *Escherichia coli* and *Klebsiella pneumoniae* isolates in JUSH, Southwest of Ethiopia.

Antibiotics	Total R (*N*%)	ESBL-positive (*n* = 17) (*N*%)		ESBL-negative (*n* = 57) (*N*%)		*p* value
	R	R	S	R	S	
Cefotaxime	18 (24.3%)	17 (100%)	0	1(1.8%)	56 (98.2%)	0.001
Ceftriaxone	17 (23.0%)	17 (100%)	0	0	57 (100%)	0.001
Ceftazidime	16 (21.6%)	12 (70.6%)	5 (29.4%)	4 (7.0%)	53 (93.0%)	0.001
AMC	27 (36.5%)	14 (82.4%)	3 (17.6%)	13 (22.8%)	44 (77.2%)	0.001
Cephalothin	53 (71.6%)	17 (100%)	0	36 (63.2%)	21 (36.8%)	0.051
Ampicillin	74 (100%)	17 (100%)	0	57 (100%)	0	—
Gentamicin	17 (23%)	11 (64.7%)	6 (35.3%)	6 (10.5%)	51(89.5%)	0.001
Amikacin	7 (9.5%)	4 (23.5%)	13 (76.5%)	3 (5.3%)	54 (94.7%)	0.024
NA	38 (51.4%)	13 (76.5%)	4 (23.5%)	25 (43.9%)	32 (56.1%)	0.001
CIP	24 (32.4%)	13 (76.5%)	4 (23.5%)	11 (19.3%)	46 (80.7%)	0.001
Norfloxacin	23 (31.1%)	13 (76.5%)	4 (23.5%)	10 (17.5%)	47 (82.5%)	0.001
SXT	41 (55.4%)	14 (82.4%)	3(17.6%)	27 (47.4%)	30 (52.6%)	0.001
Tetracycline	45 (60.8%)	14 (82.4%)	3 (17.6%)	31 (54.4%)	26 (45.6%)	0.021
C	30 (40.5%)	12 (70.6%)	5 (29.4%)	18 (31.6%)	39 (68.4%)	0.004
Imipenem	0	0	17 (100%)	0	57 (100%)	—

R: resistant, S: sensitive, AMC: amoxicillin-clavulanic acid, NA: nalidixic acid, CIP: ciprofloxacin, SXT: trimethoprim-sulfamethoxazole, C: chloramphenicol.

**Table 4:** Resistance profiles of isolates from healthcare-associated versus true community-acquired in JUSH, Southwest of Ethiopia.

Antibiotics	Total R (*N*%)	Healthcare-associated		Community-acquired		*p* value
	R	R	S	R	S	
Cefotaxime	18 (24.3%)	9 (36%)	16 (64%)	9 (18.4%)	40 (81.6%)	0.168
Ceftriaxone	17 (23.0%)	9 (36%)	16 (64%)	9 (18.4%)	40 (81.6%)	0.168
Ceftazidime	16 (21.6%)	9 (36%)	16 (64%)	7 (14.3%)	42 (85.7%)	0.041
AMC	27 (36.5%)	10 (40%)	15 (60%)	17 (36.7%)	32 (65.3%)	0.799
Cephalothin	53 (71.6%)	18 (72%)	7 (28%)	35 (71.4%)	14 (28.6%)	1.000
Ampicillin	74 (100%)	25(100%)	0	49 (100%)	0	—
Gentamicin	17 (23%)	9 (36%)	16 (64%)	8 (16.3%)	41(83.7%)	0.080
Amikacin	7 (9.5%)	4 (16%)	21 (84%)	3 (6.1%)	46 (93.9%)	0.217
NA	38 (51.4%)	14 (56%)	11 (44%)	24 (49%)	25 (51%)	0.628
CIP	24 (32.4%)	9 (36%)	16 (64%)	15 (30.6%)	34 (69.4%)	0.793
Norfloxacin	23 (31.1%)	9 (36%)	16 (64%)	14 (28.6%)	35 (71.4%)	0.793
SXT	41 (55.4%)	15 (60%)	10 (40%)	26 (53.1%)	23 (46.9%)	1.000
Tetracycline	45 (60.8%)	14 (56%)	11 (44%)	31 (54.4%)	18 (45.6%)	0.610
C	30 (40.5%)	14 (56%)	11 (44%)	16 (32.6%)	33 (67.4%)	0.079
Imipenem	0	0	25 (100%)	0	49 (100%)	—

R: resistant, S: sensitive, AMC: amoxicillin-clavulanic acid, NA: nalidixic acid, CIP: ciprofloxacin, SXT: trimethoprim-sulfamethoxazole, C: chloramphenicol.

**Table 5:** Frequency of multidrug resistance pattern of ESBL-producing isolates from community-onset UTI patients in JUSH, Southwest of Ethiopia.

Antibiotic classes	MDR rate (*N* (%))
Beta-lactams + SXT, T	14 (82.4%)
Beta-lactams + SXT, T, NA	13 (76.5%)
Beta-lactams + SXT, T, NA, CIP, GEN	11 (64.7%)
Beta-lactams + SXT, T, NA, CIP, GEN, C	9 (52.9%)
Beta-lactams + SXT, T, NA, CIP, GEN, AK, C	4 (23.5%)

Beta-lactams: (ampicillin, cephalothin, amoxicillin-clavulanic acid, cefotaxime, ceftazidime, and ceftriaxone), GEN: gentamicin, AK: amikacin, CIP: ciprofloxacin, SXT: trimethoprim-sulfamethoxazole, T: tetracycline, NA: nalidixic acid, C: chloramphenicol.

## Data Availability

All the data supporting our findings were incorporated within the manuscript. Raw data can be presented by the principal investigator upon reasonable request.
